# A long-term 0.01° surface air temperature dataset of Tibetan Plateau

**DOI:** 10.1016/j.dib.2018.08.107

**Published:** 2018-08-29

**Authors:** Lirong Ding, Ji Zhou, Xiaodong Zhang, Shaomin Liu, Ruyin Cao

**Affiliations:** a School of Resources and Environment, Center for Information Geoscience, University of Electronic Science and Technology of China, Chengdu 611731, China; b Faculty of Geographical Science and State Key Laboratory of Earth Surface Processes and Resource Ecology, Beijing Normal University, Beijing 100875, China

## Abstract

The surface air temperature (*T*_a_) dataset of the Tibetan Plateau is obtained by downscaling the China regional surface meteorological feature dataset (CRSMFD). It contains the daily mean *T*_a_ and 3-hourly instantaneous *T*_a_. This dataset has a spatial resolution of 0.01°. Its time range for surface air temperature dataset is from 2000 to 2015. Spatial dimension of data: 73°E–106°E, 40°N–23°N. The *T*_a_ with a 0.01° can serve as an important input for the modeling of land surface processes, such as surface evapotranspiration estimation, agricultural monitoring, and climate change analysis.

**Specifications Table**

**Table:** 

Subject area	*Earth and Planetary Sciences*
More specific subject area	*Atmospheric Science, Earth-Surface Processes.*
Type of data	*image*
How data was acquired	*Downscaling model*
Data format	*Raw and examples of analyzed data*
Experimental factors	
Experimental features	
Data source location	*School of Resources and Environment, Center for Information Geoscience, University of Electronic Science and Technology of China, Chengdu, China*
Data accessibility	*This data has a high temporal resolution and a medium spatial resolution; thus, this dataset is huge. In order to maximize the sharing of this data, we can only provide a link to download this dataset.*
	*Resource link:*https://pan.baidu.com/s/1SaD3gafyGJRYXjW8k8Cs7g
Related research article	*L. Ding, J. Zhou, X. Zhang, S. Liu, and R. Cao, “Downscaling of surface air temperature over the Tibetan Plateau based on DEM,” Int. J. Appl. Earth Obs. Geoinformation, vol. 73, pp. 136–147, 2018.*
	https://doi.org/10.1016/j.jag.2018.05.017

**Value of the Data**

• It can contribute to better modeling the radiation balance and energy budget and water cycle over the Tibetan Plateau.
• It can serve as an important input parameter for the modeling of land surface processes, such as surface evapotranspiration estimation.
• It can provide long-term *T*_a_ dataset with acceptable accuracy and medium spatial resolution for climate change study.

## 1 Data

As the highest plateau in the world, the Tibetan Plateau has the largest glaciers except the Arctic and Antarctic. Due to complex natural environment, the Tibetan Plateau has significant impacts on climate change of the surrounding areas and even the whole world. Because of its special geographical location and topography, the radiation balance and energy budget and water cycle examinations of the Tibetan Plateau are particularly important. Thus, the scientific communities is requiring a long-term *T*_a_ dataset with acceptable accuracy and medium spatial resolution.

**Fig. 1:**
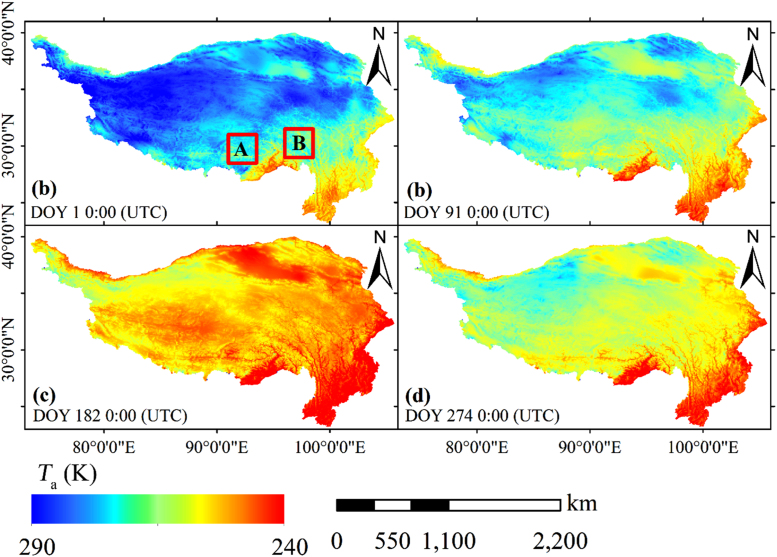
Examples of the 0.01° *T*_a_ data over the Tibetan Plate.

**Fig. 2:**
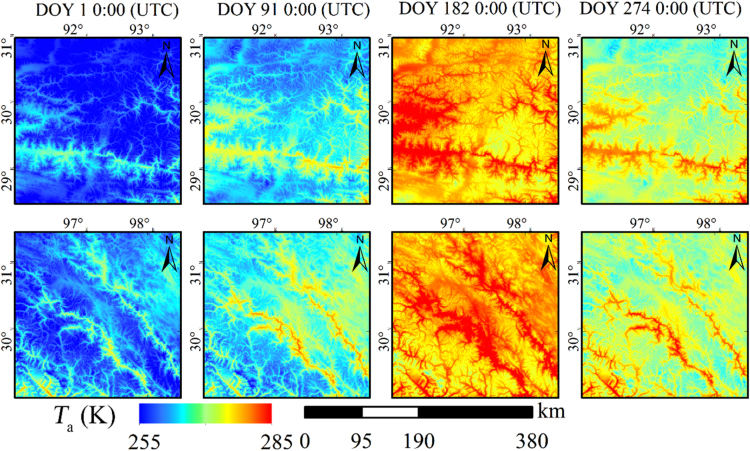
Subsets of the 0.01° *T*_a_ data.

We use the China regional surface meteorological feature dataset (CRSMFD) [1], [2] as the basis dataset. We develop a practical method to downscale the CRSMFD from 0.1° to 0.01°. The temporal resolution of this dataset is consistent with CRSMFD. It have better consistency with the ground measured *T*_a_ than original CRSMFD in Tibetan Plateau. It has higher spatial resolution than most of the current long-term *T*_a_ dataset for the Tibetan Plateau. In addition, *T*_a_ with a 0.01° resolution can reflect more spatial details of *T*_a_ when compared with the original CRSMFD. The *T*_a_ at some time is shown as an example in Fig. 1, and the 0.01° *T*_a_ of local areas is shown as an example in Fig. 2 (area A and area B are shown in Fig. 1). Thus, this dataset is able to meet the ever-increasing demand for related studies and applications.

## 2 Experimental design, materials, and methods

The linear relationship between *T*_a_ and its influencing factors, *T*_a_ can be expressed as:

(1) $$T_{a,\text{daily}}=f_{\mathrm{daily}}\left(H,X_{1}\right)=\lambda H+aX_{1}+c$$

(2) $$T_{a,\text{ins}}=f_{\mathrm{ins}}\left(H,X_{1},X_{2}\right)=\lambda H+aX_{1}+bX_{2}+c$$

where *T*_a,daily_ and *T*_a,ins_ are the daily mean and instantaneous *T*_a_ in K, respectively; *f*_daily_ and *f*_ins_ are the statistical functions for the daily mean *T*_a_ and instantaneous *T*_a_, respectively; *H*, *X*_1_, *X*_2_ are the elevation, latitude, and longitude, respectively; *λ*, *a*, and *b* are the corresponding coefficients; and *c* is the intercept. It is evident that *λ* is the lapse rate (LR) of *T*_a_ [3], [4]. Note that the longitude is not contained in Eq. (1) due to its ignorable ability in explaining daily mean *T*_a_.

**Fig. 3:**
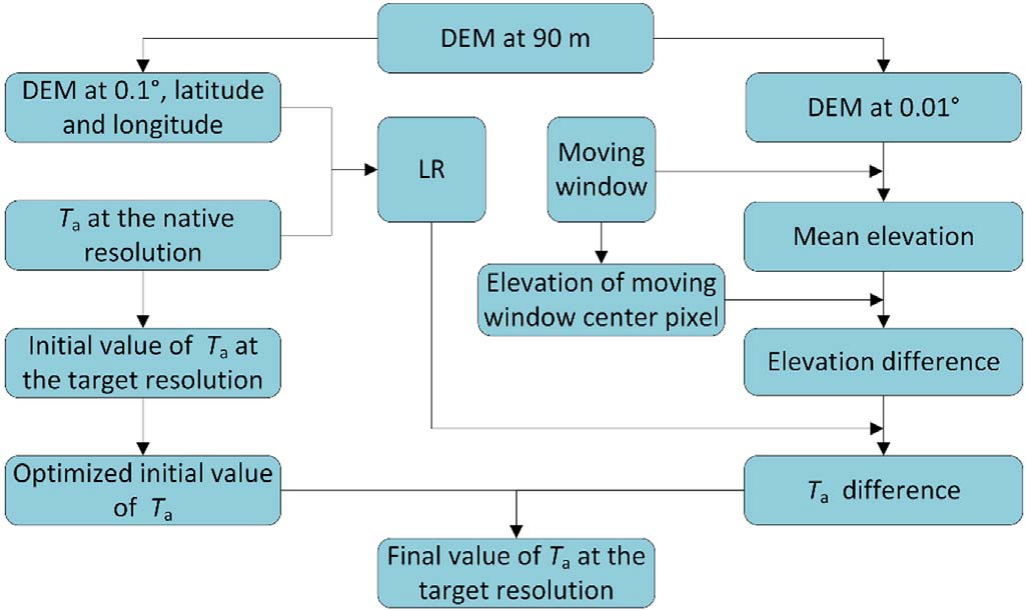
Flowchart of the method for *T*_a_ downscaling.

Based on Eqs. (1), (2), the flowchart of the proposed method for *T*_a_ downscaling is shown in Fig. 3. The first stage for *T*_a_ downscaling is to calculate LR. The DEM data at 90-m is aggregated to 0.01°. The mean elevation of the 10 × 10 pixels is calculated and used as the elevation of the pixel at 0.1° that containing these 10 × 10 pixels. The spatial distribution of LR can be divided into eight regions, i.e. Region 1: 73–90°E, 35–40°N; Region 2: 90–100°E, 35–40°N; Region 3: 100–105°E, 35–40°N; Region 4: 78–95°E, 27–35°N; Region 5: 95–100°E, 27–35°N; Region 6: 100–107.5°E, 30–35°N; Region 7: 100–105°E, 25–30°N; Region 8: 100–105°E, 23–25°N [5]. In this division scheme, each region has similar regional climatic characteristics and a range of elevation changes. This division scheme is utilized by this method. To better address the intra-annual variations of LR, the LR values of instantaneous *T*_a_ at every 3 h and the daily mean *T*_a_ on every day are calculated.

The second stage is to determine and optimize the initial value of *T*_a_ at the target resolution. The *T*_a_ value at the native resolution (i.e. 0.1°) is taken as the initial value of the pixel at the target resolution (i.e. 0.01°). At the target resolution, a moving window approach is employed to refine the initial *T*_a_ of the central pixel. For each pixel at the target resolution, the window size is set to 11 × 11 pixels and the current pixel under consideration is the center of the window. If the current pixel is on the edge of the image, the window is not complete and the existing pixels are selected. Pixels with valid *T*_a_ and elevation in the moving window are selected as valid pixels [6]. Then the mean *T*_a_ of the valid pixels in the moving window is calculated as the optimized *T*_a_ of the central pixel as follows:

(3) $${T\prime }_{a}=\frac{\sum_{i=1}^{m}T_{a-\text{initial}}\left(i\right)}{m}$$

where *T*_a_′ is optimized initial value of the *T*_a_; *T*_a-initial_(i) is the initial *T*_a_ the i-th pixel at the target resolution within the window; and *m* is the number of valid pixels in the window.

The third stage is to determine the final value of *T*_a_ at the target resolution. According Eqs. (1), (2), the *T*_a_ difference between the central pixel and the mean *T*_a_ of moving window can be expressed as:

(4) $$\Delta T_{a,\text{daily}}=\lambda (H-H_{\mathrm{win}})+a(X_{1-i}-X_{1-\mathrm{win}})$$

(5) $$\Delta T_{a,\text{ins}}=\lambda (H-H_{\mathrm{win}})+a(X_{1-i}-X_{1-\mathrm{win}})+b(X_{2-i}-X_{2-\mathrm{win}})$$

where Δ*T*_a,daily_ and Δ*T*_a,ins_ are daily mean *T*_a_ difference and instantaneous *T*_a_ difference in K; *H*, *X*_1−*i*_ and *X*_2−*i*_ are the elevation, latitude, and longitude of the central pixel of the moving window, respectively; *H*_win_, *X*_1−win_ and *X*_2−win_ are the mean elevation, latitude, and longitude of the moving window, respectively. *X*_1−*i*_ and *X*_1−win_, *X*_2−*i*_, and *X*_2−win_ can be considered to be approximately equal. Thus, Eqs. (4), (5) can be simplified as:

(6) $$\Delta T=\lambda (H-H_{\mathrm{win}})$$

where Δ*T* is the *T*_a_ difference in K.

Then the final *T*_a_ of the central pixel is:

(7) $$T_{a}=T_{a}^{\prime }+\Delta T$$

Finally, the 0.01° *T*_a_ data of Tibetan Plateau was obtained by this downscaling method.
